# Supplementary material for: Structure and evolution of the Fam20 kinases
Source: Nat Commun. 2018 Mar 23;9:1218. doi: 10.1038/s41467-018-03615-z (PMC5865150; doi:10.1038/s41467-018-03615-z)
Supplement: Supplementary file 1 — Supplementary Information(PDF 4442 kb) [file 41467_2018_3615_MOESM1_ESM.pdf]

Supplementary Information for

Structure and evolution of the Fam20 kinases

Zhang et al.

This file includes:

Supplementary Figures 1-8
Supplementary Tables 1-2

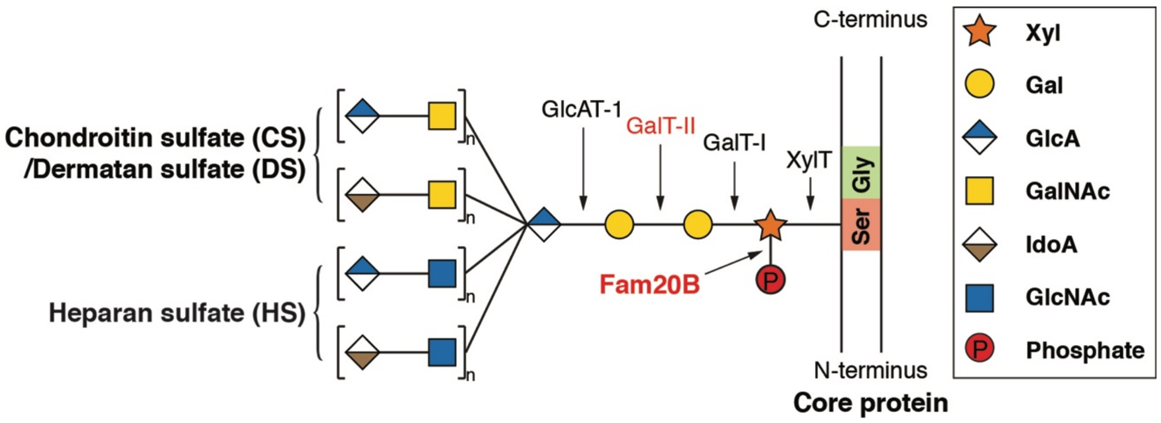

Supplementary Figure 1. A schematic of proteoglycan structures and enzymes involved in the biosynthesis of the tetrasaccharide linkage.

The biosynthesis of CS/DS and HS proteoglycans requires the formation of a tetrasaccharide linker, established by the sequential actions of xylosyltransferase (XylT), galactosyltransferase I (GalT-I), galactosyltransferase II (GalT-II), and glucuronyltransferase I (GlcAT-I). During this process, Fam20B recognizes the Gal-Xyl disaccharide and phosphorylates the Xyl at the C2 hydroxyl position, which is important for priming the activity of GalT-II.

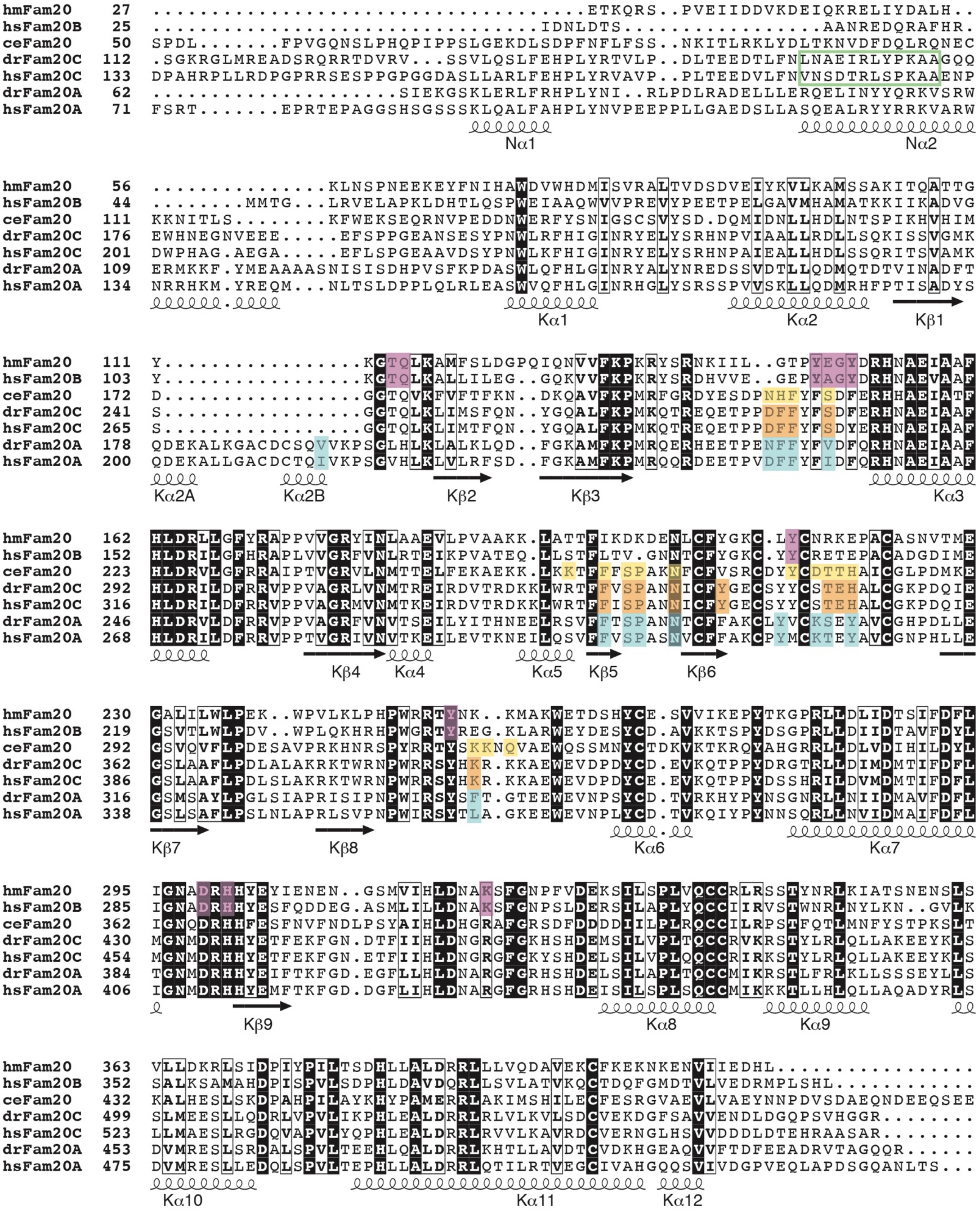

Supplementary Figure 2. Structure-based sequence alignment of select Fam20B, Fam20C, and Fam20A family members.

Identical residues in all Fam20 proteins are written with white bold characters in a black background. Similar residues are written with black bold characters and boxed in black. Disaccharide-binding residues in

Hydra magnipapillata Fam20 (hmFam20) and the corresponding residues in human Fam20B (hsFam20B) are shown in a magenta background. Dimer interface residues in *Caenorhabditis elegans* Fam20 (ceFam20) are shown in a yellow background. Dimer interface residues in *Danio Rerio* Fam20C (drFam20C) and the corresponding residues in human Fam20C (hsFam20C) are shown in an orange background. Fam20C-binding residues in human Fam20A (hsFam20A) and the corresponding residues in drFam20A are shown in a cyan background. The Nα2 helix in drFam20C that is critical for tetramer formation and the corresponding region in hsFam20C are highlighted using a green box. Secondary structures of hsFam20A are shown below the sequence blocks (N: N-terminal segment, K: Kinase domain).

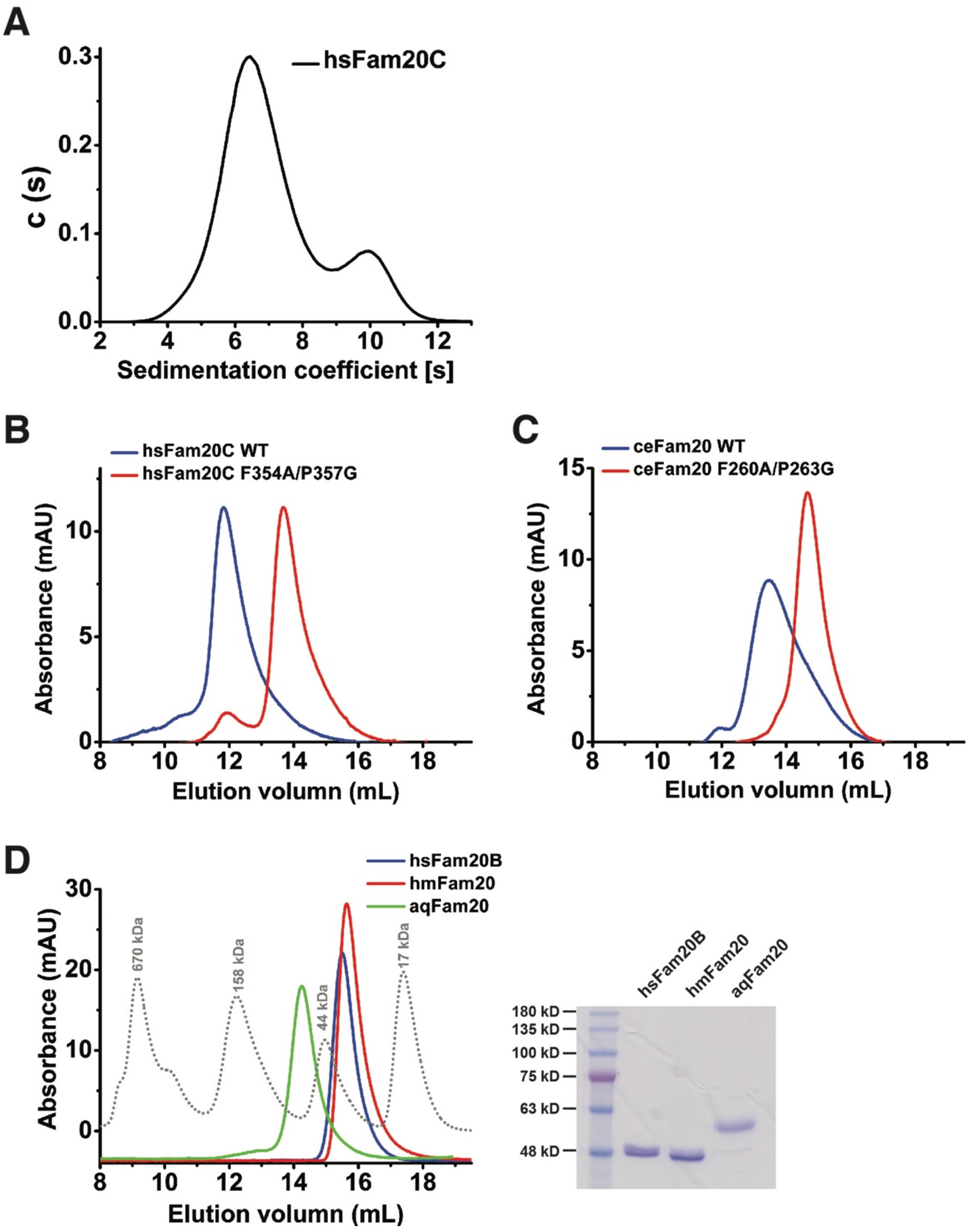

Supplementary Figure 3. The Fam20C proteins are evolutionarily conserved as dimers, while the Fam20B proteins are monomers.

(A) Sedimentation velocity analytical ultracentrifugation suggests that Fam20C is predominantly a dimer in solution at 1 mg/ml, with a sedimentation coefficient of 6.5 S.

(B) In contrast to WT Fam20, Fam20C-F354A/P357G is largely monomeric as evaluated by size exclusion chromatography.

(C) ceFam20 is a dimer in solution, while a F260A/P263G mutant is a monomer.

(D) The Fam20B proteins are monomers in solution. aqFam20 (388 amino acids, see Methods) is slightly larger than hmFam20 (361 amino acids) and human Fam20B (348 amino acids), as shown by the SDS-PAGE on the right (all three proteins contain two N-linked glycosylation sites, so they migrate more slowly compared to normal proteins on the gel). Nevertheless, aqFam20 it is still a monomer as judged by the elution positions of the size exclusion molecular weight standards.

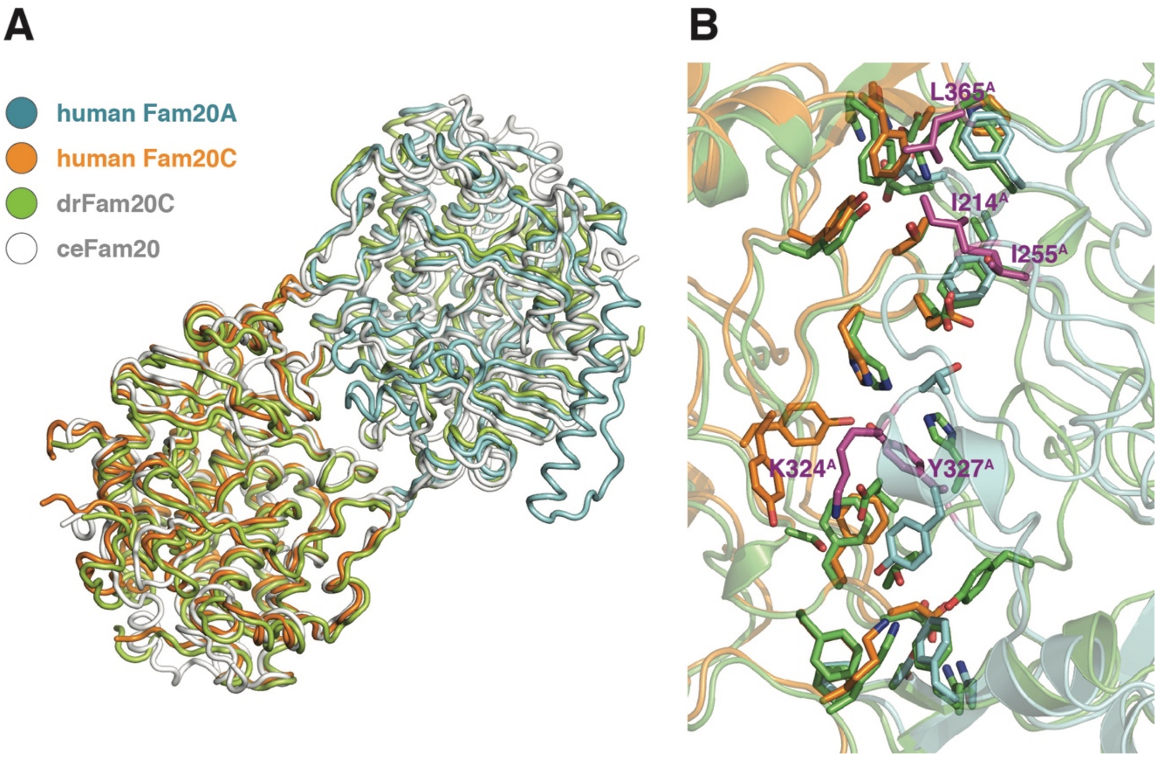

Supplementary Figure 4. Comparison of the Fam20A-Fam20C heterodimer, the drFam20C homodimer, and the ceFam20 homodimer.

(A) drFam20C and ceFam20 homodimers are superimposed on the Fam20A-Fam20C heterodimer. Fam20A is shown in cyan, Fam20C is shown in orange. The drFam20C and ceFam20 molecules are shown in green and white, respectively.

(B) Comparison of the Fam20A-Fam20C heterodimer interface and the drFam20C homodimer interface. The color scheme is the same as **(A)**. Ile214^{A}, Ile255^{A}, Leu365^{A}, Lys324^{A}, and Tyr327^{A} that are unique to Fam20A are highlighted in magenta.

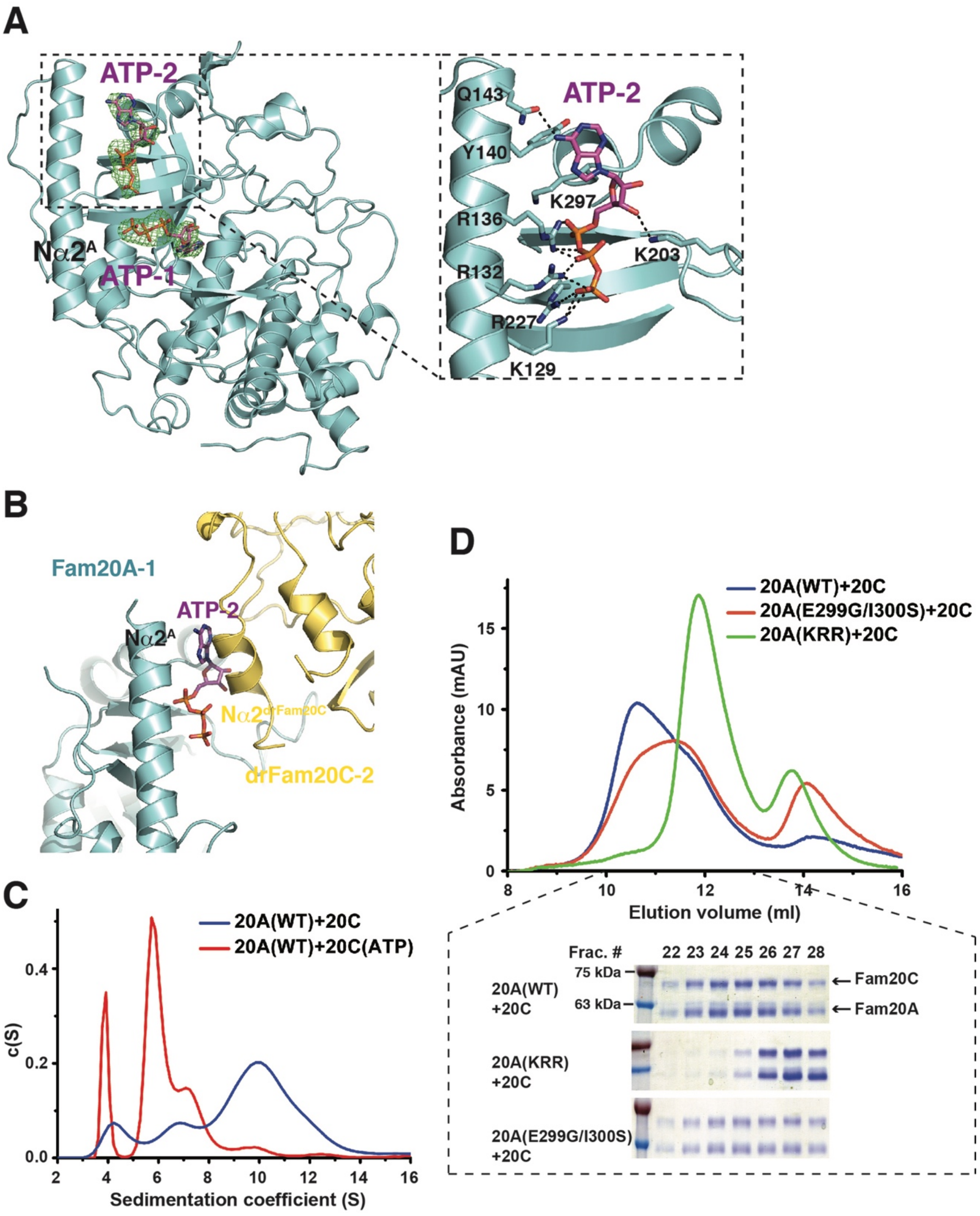

Supplementary Figure 5. Fam20A can bind a second ATP molecule, which can convert the human Fam20A-Fam20C tetramer to a dimer.

(A) Two ATP molecules (ATP-1 and ATP-2) are bound to Fam20A in the human Fam20A-Fam20C crystal structure. Green meshes represent the Fo-Fc difference electron density map (contoured at 3.0 σ) calculated before the ATP molecules were modeled. Residues in Fam20A involved in binding to ATP-2 are depicted in an enlarged image shown on the right.

(B) ATP-2 would collide with the $N\alpha 2^{drFam20C}$ helix in drFam20C-2 when the human Fam20A-Fam20C dimer is superimposed onto Fam20A-1-drFam20C-1 in the Fam20A-drFam20C tetramer, and therefore prevents tetramer formation. Only ATP-2 from the human Fam20A-Fam20C dimer, and Fam20A-1, drFam20C-2 from the Fam20A-drFam20C tetramer are shown for clarity.

(C) Sedimentation velocity analytical ultracentrifugation experiments suggest that the human Fam20A-Fam20C complex can form a homogeneous tetramer at 4 μM (with a sedimentation coefficient of 10 S). The presence of 1 mM ATP almost completely shifts the Fam20A-Fam20C complex to a dimer.

(D) The elution profiles of different Fam20A-Fam20C complexes from size exclusion chromatography. WT or mutant Fam20A were incubated with Fam20C at a molar ratio of ~1.2:1 and then passed through a Superdex 200 increase column. The running buffer contained 20 mM HEPES, pH 7.5, 100 mM NaCl. Fractions 22-28 correspond to elution volumes 10.0-12.8 ml (0.4 ml per fraction). Excess Fam20A monomer elutes at ~14 ml. KRR: K129A/R132A/R136A.

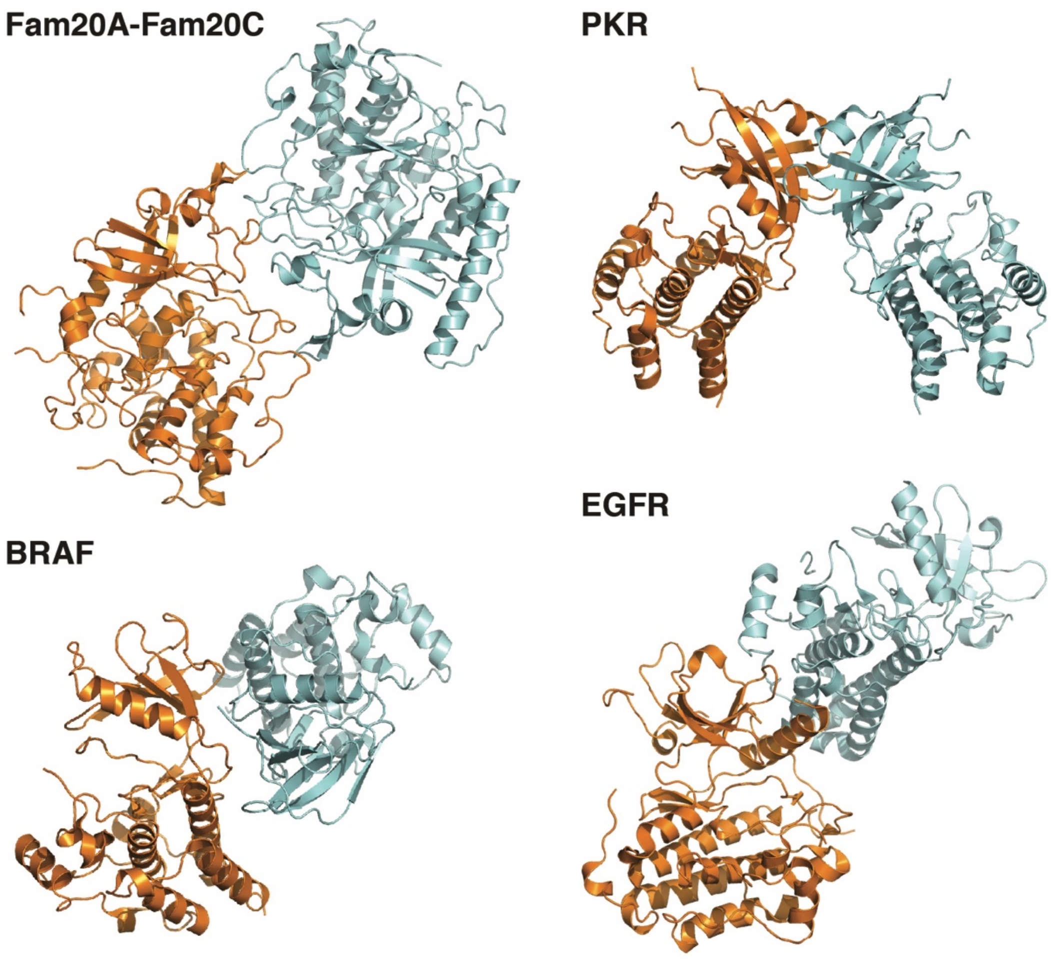

Supplementary Figure 6. The Fam20A-Fam20C, PKR, RAF, and EGFR kinases form diverse homo/heterodimers that lead to kinase activation.
Fam20C forms a reversed face-to-face homodimer or heterodimer with Fam20A. PKR and other eIF2α kinases form back-to-back dimers (illustrated by the structure of PKR, PDB ID 2A1A [http://dx.doi.org/10.2210/pdb2A1A/pdb]). The RAF family kinases form side-to-side dimers (illustrated by the structure of BRAF, PDB ID 1UWH [http://dx.doi.org/10.2210/pdb1UWH/pdb]). The EGFR family kinases form asymmetric head-to-tail dimers (illustrated by the structure of EGFR, PDB ID 2GS6 [http://dx.doi.org/ 10.2210/pdb2GS6/pdb]).

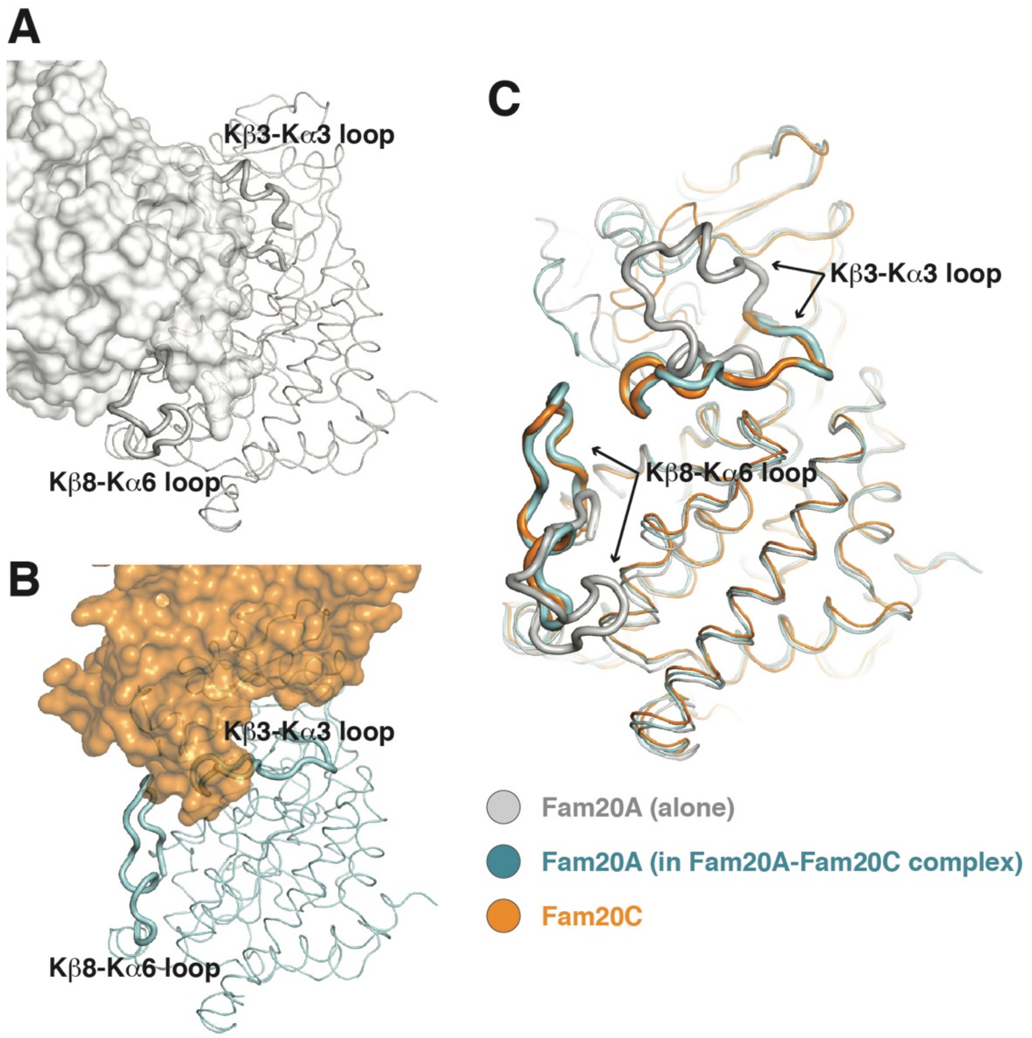

Supplementary Figure 7. Two loops in Fam20A undergo large conformational changes when Fam20A complexes with Fam20C.
(A) Structure of the Fam20A homodimer (PDB ID: 5WRR [http://dx.doi.org/10.2210/pdb5WRR/pdb]). One protomer is shown as a ribbon diagram, with the Kβ3-Kα3 and Kβ8-Kα6 loops shown as thick ribbons. The other protomer is shown as a surface representation.
(B) Structure of the Fam20A-Fam20C heterodimer. Fam20A is shown in cyan, with the Kβ3-Kα3 and Kβ8-Kα6 loops shown as thick ribbons. Fam20C is shown in orange.
(C) Structural Superposition of Fam20A (gray), Fam20A in the Fam20A-Fam20C complex (cyan), and Fam20C in the Fam20A-Fam20C complex (orange). When engaged with Fam20C, the Kβ3-Kα3 and Kβ8-Kα6 loops of Fam20A undergo large conformational changes and become Fam20C-like.

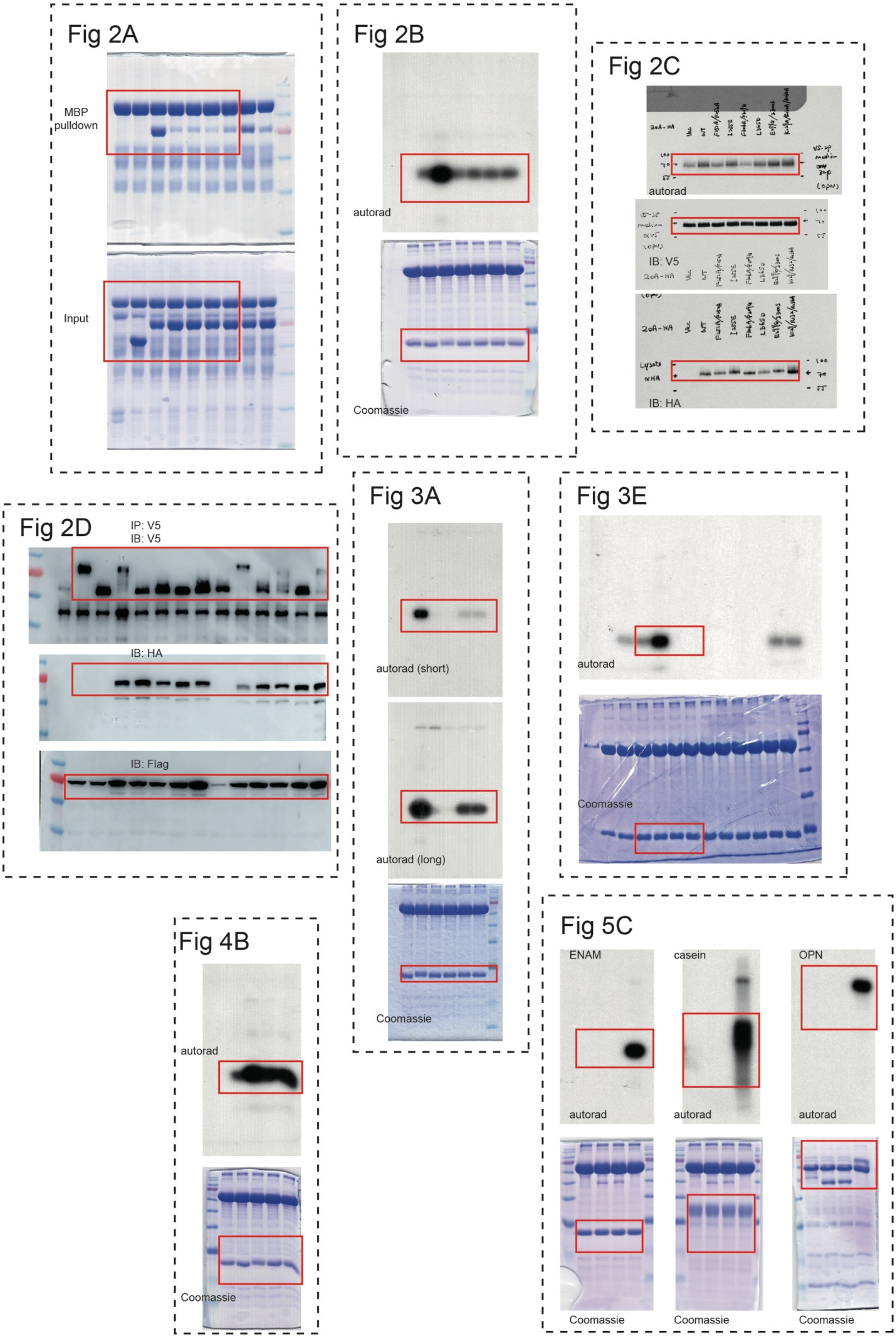

Supplementary Figure 8. Full blots and gels for results presented in the main paper.

Supplementary Table 1. List of primers used in this study.

	Forward	Reverse
aqFam20 (residues 31-418)	GCAGATCTGAAGAGCCCACACAGAACACAGTC	GCCTCGAGCTAAAACTGTGGCCACTTATCAAAGAGAACG
hmFam20 (residues 55–415)	GCGGATCCCACAAGCTCAACTCCCCTAATGAG	GCCTCGAGTCACAGATGGTCCTCA
hsFam20B (residues 55-402)	GCGGATCCAAGCTGGACCATACCTTGCAGT	GCCTCGAGCTAGTCTTCCACCAGTACTGTGTCCA
hsFam20C (residues 63-584)	GCAGATCTGTTCGGGGCCGCCCCGGG	GCCTCGAGCTACCTCGCCGAGGCGGCTC
drFam20C (residues 124-560)	GCAGATCTCGGCAGAGGCGCACTGAT	GCGAATTCCTACCTGCCTCCGTGCACTGA
Fam20A-K129A/R132A/R136A	TGTACATCTTGTGTGCCCTGTTCCAGGCGGCCACCGCCCTCCGGTAATACC	GGTATTACCGGAGGGCGGTGGCCGCCTGGAACAGGGCACACAAGATGTACA
Fam20A-F251A/F252A	AGACACCAGTGGACGCTGCTTACTTCATTGACTTTCA	TGAAAGTCAATGAAGTAAGCAGCGTCCACTGGTGTCT
Fam20A-I255E	GACTTCTTCTACTTCGAAGACTTTCAGAGAC	GTCTCTGAAAGTCTTCGAAGTAGAAGAAGTC
Fam20A-E299G/I300S	GAGGTCACCAAGAATGGATCTCTGCAGAGTGTTTT	AAAACACTCTGCAGAGATCCATTCTTGGTGACCTC
Fam20A-F306A/P309G	AGAGTGTTTTCGCTGTCTCTGGAGCGAGCAACGT	ACGTTGCTCGCTCCAGAGACAGCGAAAACACTCT
Fam20A-L365D	ATCCGCTCCTACACAGACGCAGGAAAAGAGGAG	CTCCTCTTTTCCTGCGTCTGTGTAGGAGCGGAT
Fam20C-G280R	ACCTTCCAGAATTACCGGCAAGCGCTGTTCAAA	TTTGAACAGCGCTTGCCGGTAATTCTGGAAGGT
Fam20C-F299AF300A	GAGACACCCCCTGACGCTGCTTATTTCTCTGACTAC	GTAGTCAGAGAAATAAGCAGCGTCAGGGGGTGTCTC
Fam20C-F354A/P357G	CTCTGGAGGACCTTCGCCATCTCTGGAGCCAACAACATCTGC	GCAGATGTTGTTGGCTCCAGAGATGGCGAAGGTCCTCCAGAG
Fam20C-H375Y	TACTGCTCCACGGAGTACGCCCTGTGCGGGAAG	CTTCCCGCACAGGGCGTACTCCGTGGAGCAGTA
Fam20C-E374S/H375T	TACTACTGCTCCACGTCGACCGCCCTGTGCGGGAAG	CTTCCCGCACAGGGCGGTCGACGTGGAGCAGTAGTA
Fam20C-G379E	AGCACGCCCTGTGCGAGAAGCCAGACCAGAT	ATCTGGTCTGGCTTCTCGCACAGGGCGTGCT
ceFam20-F260A/P263G	CTCAAGAAGACTTTCGCCTTCTCTGGAGCCAAAAACTTTTGC	GCAAAAGTTTTTGGCTCCAGAGAAGGCGAAAGTCTTCTTGAG
Fam20B-T106L	TGGGTTATAAAGGGCTACAGCTGAAAGCCTTA	TAAGGCTTTCAGCTGTAGCCCTTTATAACCCA
Fam20B-Q107A	GTTATAAAGGGACAGCGCTGAAAGCCTTACTG	CAGTAAGGCTTTCAGCGCTGTCCCTTTATAAC
Fam20B-G140D	GGAACCGTATGCTGATTATGATAGACACAATG	CATTGTGTCTATCATAATCAGCATACGGTTCC
Fam20B-Y242F	ATGGGGCAGGACTTTCCGAGAAGGCAAATTG	CAATTTGCCTTCTCGGAAAGTCCTGCCCCAT
Fam20B-D289A	GATTGGCAATGCTGCCCGCCATCACTATGAG	CTCATAGTGATGGCGGGCAGCATTGCCAATC
Fam20B-H291A	GCAATGCTGACCGCGCTCACTATGAGAGCT	AGCTCTCATAGTGAGCGCGGTCAGCATTGC
Fam20B-K312R	TCTTGATAATGCCAGAAGCTTTGGGAACCC	GGGTTCCCAAAGCTTCTGGCATTATCAAGA

Supplementary Table 2. Codon optimized DNA sequence of hmFam20 (encoding residues 24-415).

TCCAAACTGGAAACAAAGCAGCGGAGCCCCGTGGAGATTATTGACGATGTGAAAGATGAGATTCAGA<br>AGAGGGAACTGATTTACGATGCCCTGCACAAGCTCAACTCCCCTAATGAGGAAAAAGAGTACTTCAA<br>CATTCACGCATGGGACGTGTGGCATGATATGATCTCCGTCAGGGCCCTGACCGTGGACAGCGATGTCG<br>AAATTTACAAGGTGCTGAAAGCAATGTCCAGCGCCAAGATCACCCAGGCTACCACTGGCTATAAGGG<br>AACTCAGCTGAAAGCAATGTTCAGCCTCGACGGCCCTCAGATCCAGAACGTGGTCTTCAAGCCAAAG<br>AGGTACTCTAGAAACAAGATCATCCTGGGTACTCCATACGAGGGCTATGACCGGCACAACGCTGAAA<br>TCGCCGCTTTCCATCTGGATCGTCTGCTCGGATTTTACCGGGCACCCCCTGTGGTCGGTCGTTATATTA<br>ACCTGGCAGCAGAGGTGCTCCCAGTCGCTGCAAAGAAACTGGCCACAACCTTCATCAAGGACAAAGA<br>TGAGAATCTGTGCTTTTACGGAAAGTGCCTCTATTGTAACAGAAAAGAACCCGCCTGTGCTTCAAATG<br>TGACCATGGAGGGAGCACTCATCCTGTGGCTCCCAGAAAAGTGGCCTGTCCTGAAACTCCCACACCCC<br>TGGCGCAGGACATACAACAAGAAAATGGCTAAGTGGGAGACCGACTCACATTACTGCGAGAGTGTGG<br>TCATTAAGGAACCATATACTAAAGGTCCCAGACTGCTCGACCTGATTGATACATCCATCTTCGACTTT<br>CTGATCGGCAATGCCGATCGTCACCATTACGAGTATATTGAGAACGAAAATGGCTCTATGGTGATCCA<br>CCTGGACAACGCTAAGTCATTCGGAAATCCCTTTGTGGATGAAAAGAGCATTCTGTCTCCTCTCGTCC<br>AGTGCTGTCGGCTGCGCTCTTCAACTTACAACCGCCTCAAGATCGCTACAAGCAACGAGAATAGTCTG<br>TCCGTGCTGCTCGACAAGAGGCTCAGTATCGATCCTATTTACCCAATCCTGACCTCCGACCATCTGCTC<br>GCCCTGGATAGACGTCTGCTCCTGGTGCAGGACGCTGTGGAGAAGTGCTTTAAGGAGAAAAACAAGG<br>AGAATGTGATTATTGAGGACCATCTGTGA
